# Power of Good Humor

**Published:** 1872-08

**Authors:** 


### POWER OF GOOD HUMOR.

THE effervescing, the overflowing, the irrepressible good humor of Franklin made a way for him wherever he went; in Christian America, in the halls of Congress, in infidel France, in the age of her disgraceful and inhuman and bloody revolution, Franklin was alike successful in carrying out his political purposes. What a life lights up in every eye, how instinctively a private company or a public crowd make a way for the man who is known to be full of fun, to be a jovial soul! But good nature has its foundation in good health, in an industrious, temperate life, with a loving heart at the bottom of it all.

